# Supplementary material for: Paternity Assignment in White Guinea Yam (Dioscorea Rotundata) Half-Sib Progenies from Polycross Mating Design Using SNP Markers
Source: Plants (Basel). 2020 Apr 19;9(4):527. doi: 10.3390/plants9040527 (PMC7238154; doi:10.3390/plants9040527)
Supplement: Supplementary file 1 [file plants-09-00527-s001.pdf]

Table S1. Contributions of the female (TDrOjuiyawo**)** and putative male (TDr9501932, TDr9902789 and TDr9902607) parents to progeny of family TDr1692

Progeny	TDr9501932	TDr9902789	TDr9902607	TDrOjuiyawo*	Intercept	Status
TDrOjuiyawo_39	0.00	0.00	**61.90**	9.14	23.70	Hybrid
TDrOjuiyawo_67	0.00	9.24	**60.91**	7.43	22.41	Hybrid
TDrOjuiyawo_91	**58.87**	0.00	12.46	4.86	23.80	Hybrid
TDrOjuiyawo_132	**59.17**	0.00	1.66	10.51	28.66	Hybrid
TDrOjuiyawo_159	0.00	21.03	**41.04**	8.49	22.37	Hybrid
TDrOjuiyawo_176	**63.20**	0.00	0.00	12.39	24.41	Hybrid
TDrOjuiyawo_52	**63.51**	0.00	8.30	2.28	25.91	Hybrid
TDrOjuiyawo_71	0.00	15.17	**47.21**	7.65	29.97	Hybrid
TDrOjuiyawo_93	**62.56**	0.00	1.84	11.68	23.91	Hybrid
TDrOjuiyawo_143	**63.40**	0.00	0.00	12.69	23.91	Hybrid
TDrOjuiyawo_160	0.00	9.64	**55.21**	7.28	25.83	Hybrid
TDrOjuiyawo_177	**59.71**	0.00	0.00	9.05	31.24	Hybrid
TDrOjuiyawo_53	0.00	11.01	**58.17**	3.18	27.65	Hybrid
TDrOjuiyawo_75	0.00	21.56	**42.39**	6.91	26.96	Hybrid
TDrOjuiyawo_96	0.00	15.98	**53.28**	7.17	23.57	Hybrid
TDrOjuiyawo_145	**63.35**	0.00	0.00	6.19	30.47	Hybrid
TDrOjuiyawo_163	**45.55**	0.00	12.32	6.66	33.10	Hybrid
TDrOjuiyawo_180	**64.99**	0.02	0.00	2.07	32.92	Hybrid
TDrOjuiyawo_54	0.00	10.93	**46.79**	5.86	35.90	Hybrid
TDrOjuiyawo_81	0.00	8.58	**60.74**	7.29	23.39	Hybrid
TDrOjuiyawo_97	0.00	15.00	**46.88**	15.80	21.87	Hybrid
TDrOjuiyawo_146	0.00	11.77	**61.86**	3.55	22.81	Hybrid
TDrOjuiyawo_167	**63.48**	0.00	0.00	6.39	30.13	Hybrid
TDrOjuiyawo_181	0.00	6.09	**63.39**	2.15	28.37	Hybrid
TDrOjuiyawo_17	**65.48**	0.00	0.02	7.78	26.73	Hybrid
TDrOjuiyawo_56	**64.93**	0.00	1.86	8.52	24.69	Hybrid
TDrOjuiyawo_82	0.00	5.26	**60.39**	12.09	22.26	Hybrid
TDrOjuiyawo_108	0.00	15.49	**51.73**	13.78	19.00	Hybrid
TDrOjuiyawo_150	**63.30**	0.00	5.19	4.69	26.82	Hybrid
TDrOjuiyawo_168	0.00	9.70	**46.27**	10.56	33.48	Hybrid
TDrOjuiyawo_185	**61.57**	0.00	7.97	3.36	27.10	Hybrid
TDrOjuiyawo_19	**66.39**	0.00	0.00	7.42	26.20	Hybrid
TDrOjuiyawo_60	0.00	6.07	**53.91**	6.13	29.10	Hybrid
TDrOjuiyawo_85	0.00	3.41	**59.11**	4.99	32.49	Hybrid
TDrOjuiyawo_113	0.00	14.17	**53.05**	11.40	21.38	Hybrid
TDrOjuiyawo_151	**59.84**	0.00	7.26	2.54	28.34	Hybrid
TDrOjuiyawo_169	0.00	4.72	**56.62**	8.68	29.98	Hybrid
TDrOjuiyawo_162	**66.10**	0.00	0.51	8.86	24.53	Hybrid
TDrOjuiyawo_33	**64.12**	0.00	0.00	8.00	27.88	Hybrid
TDrOjuiyawo_61	0.00	14.74	**48.60**	11.21	25.45	Hybrid
TDrOjuiyawo_88	0.00	11.61	**53.53**	8.76	24.11	Hybrid
TDrOjuiyawo_116	0.00	0.00	0.00	10.72	26.30	**Outlier**
TDrOjuiyawo_155	0.00	14.68	**45.87**	13.94	25.51	Hybrid
TDrOjuiyawo_171	0.00	21.48	**51.25**	4.47	22.80	Hybrid
TDrOjuiyawo_35	0.00	12.15	**60.46**	2.79	24.60	Hybrid
TDrOjuiyawo_64	**61.97**	0.00	4.18	8.92	24.33	Hybrid
TDrOjuiyawo_89	0.00	9.22	**55.03**	8.51	24.78	Hybrid
TDrOjuiyawo_129	**63.48**	0.00	0.00	8.95	27.57	Hybrid
TDrOjuiyawo_157	**58.22**	0.00	5.29	5.54	30.94	Hybrid
TDrOjuiyawo_173	0.00	18.19	**49.11**	7.31	25.40	Hybrid

* wrong female sample collected making the intercept value higher than female of all progeny developed
